# The Potential of Dyslexic Individuals in Communication Design Education

**DOI:** 10.1155/2007/327530

**Published:** 2008-03-05

**Authors:** Muzaffer Çorlu, O&gcaron;uzhan Özcan, Ümran Korkmazlar

**Affiliations:** ^1^Department of Music and Performing ArtsYildiz Technical UniversityIstanbulTurkey; ^2^Department of Communication DesignYildiz Technical UniversityIstanbulTurkey; ^3^Department of Child and Adolescent PsychiatryFaculty of MedicineYildiz Technical UniversityIstanbulTurkey

**Keywords:** Dyslexia, creativity, design education, communication design, information design

## Abstract

If dyslexic individuals have the ability to express themselves in different ways, particularly in the field of modern graphic design, would they be a favoured group in creating the extraordinary and outstanding ideas that are required in communication design? The study group consisted of 20 primary school dyslexics between ages of 7–12 and 20 non-dyslexics serving as a control group. A jury with four specialists evaluated the drawings gathered from the 40 participants. Even though we might not say surely that the dyslexics are the best possible candidates for communication design education, based on the statistical results we have concluded that they should be among the potential candidates for both general communication design education and for more specific minor study areas such as icon design.

## 1. Introduction

Dyslexic individuals have serious difficulty in reading, writing, verbal expression and drawing. Moreover, their school performance is not in accordance with their intellectual capacity [1]. In our experience the handwriting of dyslexics generally appears different from that of non dyslexics. Their writings are usually slanted. Letters are often lined up like mirror images. Letters are piled up on one side of the paper or, when there is no more space on a line, they are added at the end of the next line. (Fig. 1a).

Examining the notebooks of a group of primary school dyslexic children, we noted writing styles with different characteristics than those of non-dyslexic children, most likely because of the perceptual differences between the two groups. When we asked them to explain these differences, a typical reply was: “The ones that are different are not ours, but the others.”

In society, as well as in medicine, psychology and education, it is believed that the performance of these individuals should be improved and considerable effort is made to achieve this goal. However, in the case of communication design, this situation can be considered differently. For example, in order to emphasize the meaning of the text, a professional graphical designer utilizes different angles, type sizes, characters and even irrational layouts just as dyslexics do (Fig. 1b).

If these dyslexic individuals generally have the ‘ability’ (or rather ‘tendency’) to express ideas and perceive concepts differently, could they then be considered as an advantageous group that is able to explore the ‘remarkable and rather creative interpretations’ that are valuable requirements for communication design?

This study has been planned and designed to answer this question, with the possibility that some key points might be discovered indicating that dyslexics are potentially good communication designers. Positive answers could then yield new scientific data that would be important both for better understanding dyslexic individuals and for the development of communication design education.

ISSN 0953-4180/07/$17.00 © 2007 – IOS Press and the authors. All rights reserved a. A dyslexic writing example. b. Professional design example.

Fig. 1. (1a) A dyslexic writing example. (1b) Professional design example.

## 2. Recent studies on dyslexics' creativity

Over the last two decades, several studies using college students have examined the link between dyslexia and creativity in art and the tendency of dyslexics to choose art education.

In an important study concerning dyslexia and creativity done by LaFrance, 30 dyslexics and control participants were examined. The results were not statistically significant, but this research concluded with the result that dyslexics were more open to alternatives [2].

In an intriguing anecdotal observation, Everatt and his colleagues conducted another important study where they investigated the creativity of dyslexic teenagers. When compared to 16 control participants, 17 teenaged dyslexics did not show higher scores in this study. However in the same study, adult dyslexics received higher scores than control participants [3].

Another important study Karolyi et al., compares middle and high school students with dyslexia ($n = 29$, 17 male and 12 females) from an independent school for students with dyslexia to public school students ($n = 35$, 18 males and 17 females) for global processing of visual-spatial information [4]. The results showed that individuals with dyslexia were faster than controls at recognizing impossible figures. These results suggest that individuals with dyslexia have superior global visual-spatial processing ability.

Wolff and Lundberg recently performed studies in this field, which clearly indicated that a prevalence of dyslexia in art academy students was higher than in non-art university students [5]. Depending on self reports, the prevalence of dyslexia in art students was 15% while it was only 1% in economics students.

From the point-of-view of art and design, some studies show remarkable facts: Not only Da Vinci and Picasso, but many other artists suffered from learning disabilities when they were in primary school [6]. In their book "Picasso", Berdenac and Bouchet [7] declared that Picasso was able to shape the light and shadow in a very short time although he was unable to read, write and calculate properly [7]. It is obvious that unusually talented people with dyslexia such as Da Vinci, Rodin and Picasso might not be well educated with normal school material and methods. Although some studies indicated that problems like 'learning phobias' and 'absenteeism' also occur in these people's lives, dyslexics could be successful in areas needing creativity [8,9].

In order to provide a better understanding of students' self-perception of their adaptation difficulties, Heiman and Kariv [10] argued that individualized education could help dyslexic students' coping strategies. In the same study, in order to enhance effective learning and to develop self esteem, it was suggested that these people might be directed towards fields like sports, graphic design, and computer sciences.

In Ott's book *Dyslexia*, it was mentioned that dyslexic people did not prefer to continue their education in those fields where essay writing was important [11]. This could be because of their orthographic difficulty as well. However Zdziensky reported in 1996 that 24% of 109 dyslexic university students have chosen design schools [12].

In the literature referenced above studies make comparisons between non-dyslexics and dyslexics. However these dyslexics have been subject to treatment to overcome their difficulties and therefore could have

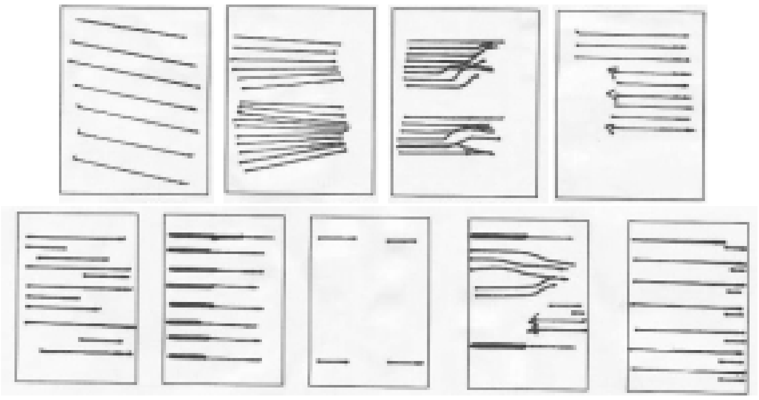

Fig. 2. Graphical display of typical writing styles of dyslexics.

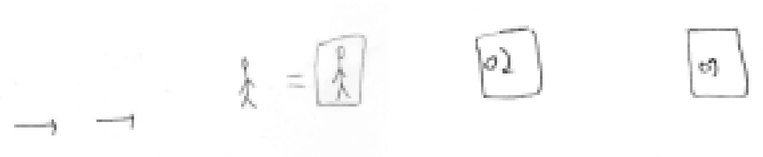

a. b. c. d.

Fig. 3. Examples of instead of to the answers given by dyslexics to to go forward, to copy, to open, and to close.

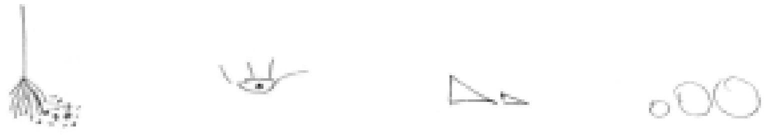

a. b. c. d.

Fig. 4. Examples of instead of to the answers given by dyslexics to to clean, to look, to reduce, and to enlarge.

changed or developed some new adaptive behaviours to cope with their disabilities. This leaves open the possibility of examining the specific artistic potential of dyslexic people who have not undergone medical and psychological treatment.

Although the literature describes possible advantages of dyslexia, it does not clarify in which exact field they are advantageous. To be able to point out that dyslexic individuals could be potentially good at communication design education, more extensive empirical experiments are still needed.

## 3. Method

This study compares dyslexic students in primary school with a control group concerning communication design abilities. Participants were tested directly through a test, and afterwards, the tests were evaluated by a group of judges with extensive experience in evaluating communication design performance. During the test, 20 dyslexics (test group) and 20 non-dyslexics (control group), a total of 40 children, were asked to make drawings that were supposed to describe the 20 given "actions". The jury members, who for the last 5 years have been evaluating communication design entrance "special talent" examinations -which are based on drawing- at Yildiz Technical University (Istanbul), subsequently evaluated the answers given by the participants. They evaluated and scored the test papers without knowing which paper belonged to which group. Every member of the jury evaluated each paper individually without knowing other jury members thoughts and remarks, and therefore they could not be affected by each other.

Each participant's answers were scored as successful or unsuccessful, purporting respectively that the participant was considered suitable or unsuitable for communication design education. Moreover, the percentage of navigational sign usage and different symbols for opposition were taken into consideration while these are important creativity criteria for Communication Design field.

Finally for each individual, it was determined how many of their 20 responses were found successful and then they were scored a cumulative grade from 0 to 100. As we had 20 people in each group we used the non-parametric Mann Whitney U test.

For this study, a more extensive version of the aptitude exam being used at the Yildiz Technical University Communication Design Department for student selection was developed as the testing tool. The test participants were asked to respond to 20 different conceptual terms by drawing them on an A4 sized blank page. These verbs included interaction terms used for computer interface design (to open, to close, to enlarge, to reduce, to go forward, to return, to erase, to copy, to carry, to call) as well as terms for everyday actions that could be used in communication design (to listen, to look, to talk, to draw, to stop, to eat, to smell, to drink, to look for, to ask for help).

The words belonging to the computer interaction terms in question were specially selected from "operational" and "editing" processes in the computer field. The scope of the test was expanded by the addition of new everyday actions to these terms. In this way, the expressional varieties of the participants were able to be measured by taking into consideration their tendency to express themselves only by drawing, as well as their abstraction ability.

Drawings were thereby obtained for evaluation and drawing tests. These are one of the most important parts of evaluation and selecting criteria for Communication Design education [13].

As far as communication design candidates are concerned, characteristics such as "presenting the information visually in the most correct and easiest way, the originality of the idea and expressing the message as clearly as possible" are very important.

## 4. Participants

Dyslexic test participants were selected from a group of children who suffered in school and were diagnosed with dyslexia at the Istanbul Faculty of Medicine. They were diagnosed through psycho educational assessments: family interview, WISC-R, Gessel Developmental test, Bender-Gestalt tests, Visual additive digit span, Turkish reading and writing test, Mathematic assessment, child behaviour check list-CBCL, and informal testing. Finally dyslexics were diagnosed according to DSM-IV and ICD-10 standards. In addition, we took care to select members for the test group who had not been in any kind of treatment.

The control group had the same characteristics as the dyslexic group, except for the latter having dyslexia. The control participants were selected from several primary schools in Istanbul. The percentage of female and male participants was 50% and the average age was 9 for both groups.

The control group had the same characteristics as the dyslexic group, except for the latter having dyslexia. The percentage of female and male participants was 50% and the average age was 9 for both groups. Each group includes two left-handed participants

The main reason why the study selected newly diagnosed dyslexics from primary school as participants is that especially around those ages, they learn how to read and write and that their tendency for drawing is increased. The older ones have already been in special education for some time and oriented to a special field. Therefore, most likely they have lost their "pure dyslexic characteristics." Because of the probability that the mentioned situation could mislead the observer, those older students were not preferred for this particular study.

## 5. Results

The determined test participant profile has been evaluated with the overall criteria that have been presented in Table 1. As a result, 10 female and 10 male primary school dyslexic children were successfully compared to their non-dyslexic counterparts. All of the test participants were between 7 and 12 years old and had scored between 98–115 IQ (according to WISC-R test). The members of the dyslexic group were not undergoing any kind of medical treatment at that time.

The overall data was analyzed statistically by a two-tailed Mann-Whitney U test (Table 2). As the scores given by 4 jury members were separately and statistically evaluated, the difference between scores of the test and control group appeared to be statistically very significant for Juror 1 and not statistically significant for Jurors 2, 3 and 4. However, when the average scores

Table 1
Participant Profile

| Age | Between 7–12 (average 9). |
|---|---|
| Education | Primary school students<br>(between 1. and 5. grades) |
| Gender | Female (50%) – Male (50%) |
| Intelligence level | IQ between 98–115 |
| Psychological characteristics | With no pathological disorders and have not been on a special treatment |
| Physical characteristics | With no disorders after neurological, psychiatric, oral and eye examinations. |
| Characteristics of the parent's | At least secondary school graduates |
| Left-handedness | only two left-handed in each group |

gathered from all of the jury members were compared, the difference was found to be statistically significant in favour of the dyslexics. After the final debate between the jury members, 9 individuals were considered to be successful for the study of communication design. Out of those 9 individuals, 5 were dyslexics and 4 were non-dyslexics.

The frequent use of 'navigational sign' was evaluated and the results were again strongly in favour of dyslexics. While this frequency was 45% in the dyslexic group, it was only 5% in the control group.

70% of the test group had replied with different symbols to the questions involving opposition, like 'to open' – 'to close', 'to make bigger' – 'to make smaller' and 'to go forward' – 'to return'. For example, a dyslexic might draw an open door to signify 'to open' but might draw a window to signify 'to close.' This 70% ratio went down to 40% in the case of the control group.

The average total answering duration was 15 minutes and 20 minutes for the test and control group, respectively.

After evaluating the successful answers of both groups, the jury declared that, dyslexic ones were more imaginative and creative. However, this result is a general opinion of the jury rather than a numerical outcome.

## 6. Discussion

Although the difference between the scores of the test and control group was found as statistically significant only in the case of Juror 1, the number of successful answers was in favour of the dyslexic group in all cases. The results obtained after the debate show that 5 out of 9 successful candidates were dyslexics. The jury members discussed with each other about results after they gave point to responses independently to eliminate inter-rater unreliability. Although dyslexics have problems with drawing, the results are encouraging enough to conclude that the dyslexic group is at least not disadvantaged, since this test is based on drawing skills. LaFrance is one of the first researchers to reveal that dyslexics are more open to alternatives. Our results strongly confirm this argument, specifically in the Communication Design field.

According to the world wide accepted admission criteria to Communication Design education, the frequent use of navigational sign is an indicative distinction. Between the two groups, dyslexics choose to express actions with navigational sign far more frequently than the control group does (Table 3). This may signify that dyslexic individuals have more tendencies to use sign language. Furthermore, this tendency could also be a sign of their ability in signage, which is a special branch of Communication Design, as well as in icon design, which is integral to computer interface and way finding. In fact, Everatt and his colleagues [3] have indicated that dyslexic adults exhibit more creativity in professions which require innovative thinking and abstraction. They also have a more creative way of thinking than non-dyslexic adults.

Using different symbols to answer the questions involving opposition, like 'to open' – 'to close,' is considered a positive sign for creativity in creativity tests as indicated in the Guilford theory [14]. Later Torrance determined four creative abilities: fluency, flexibility, originality and elaboration. Accordingly, in admission examinations, this indication is considered to be important. Since, 70% of the test group had replied by different symbols to the questions involving opposition, it can be taken as another clue that Communication Design education would be suitable for them.

Compared to the group without dyslexia, dyslexics generally answer the questions more quickly. (Averages of total answering duration; Test group: 15 minutes, Control group: 20 minutes). This result could have ideas parallel to what Kalolyi et al reported in 2003 as mentioned in the Literature part. This difference could result from dyslexics' expression choices, such that they generally do not prefer to draw a sce-

Table 2
Raw scores and Mann-Whitney U test results. (Raw scores represent the number of successful answers out of 20 answers for each group.)

| Target Actions | Juror 1 | | Juror 2 | | Juror 3 | | Juror 4 | |
|---|---|---|---|---|---|---|---|---|
| | Dyslexic group | Control group | Dyslexic group | Control group | Dyslexic group | Control group | Dyslexic group | Control group |
| to open | 7 | 1 | 4 | 5 | 5 | 3 | 6 | 5 |
| to close | 6 | 1 | 3 | 3 | 2 | 4 | 5 | 3 |
| to reduce | 5 | 3 | 6 | 7 | 7 | 4 | 8 | 3 |
| to enlarge | 3 | 2 | 6 | 5 | 4 | 3 | 4 | 2 |
| to go forward | 6 | 1 | 8 | 5 | 5 | 2 | 7 | 4 |
| to return | 7 | 2 | 9 | 6 | 7 | 5 | 9 | 5 |
| to erase | 5 | 3 | 5 | 4 | 2 | 2 | 3 | 5 |
| to copy | 8 | 5 | 7 | 7 | 8 | 4 | 6 | 8 |
| to carry | 10 | 8 | 8 | 7 | 6 | 5 | 8 | 6 |
| to call | 3 | 3 | 7 | 7 | 2 | 3 | 5 | 3 |
| to listen | 11 | 5 | 11 | 5 | 7 | 5 | 3 | 4 |
| to look | 6 | 5 | 5 | 5 | 4 | 5 | 6 | 2 |
| to talk | 6 | 4 | 8 | 7 | 3 | 3 | 4 | 1 |
| to draw | 10 | 5 | 8 | 9 | 6 | 6 | 4 | 6 |
| to stop | 2 | 1 | 7 | 8 | 5 | 4 | 4 | 7 |
| to eat | 3 | 3 | 7 | 7 | 3 | 2 | 2 | 1 |
| to smell | 3 | 3 | 7 | 6 | 3 | 3 | 1 | 3 |
| to drink | 5 | 3 | 10 | 11 | 3 | 4 | 2 | – |
| to look for | 7 | 2 | 7 | 8 | 5 | 3 | 2 | 4 |
| to ask for help | 2 | 0 | 6 | 4 | 4 | 4 | 4 | 3 |
| z value | 3.25954 | | 1.17668 | | 1.39308 | | 1.1902 | |
| p value two tailed | 0.000742 | | 0.242988 | | 0.16562 | | 0.241264 | |

nario, and that they prefer writing less than the control group does. In expressing a message in the Communication Design field, it is important that visual elements are used; thusly, clear statements that can be perceived immediately are made. More importantly, during the design process, thinking and responding very rapidly are essential requirements.

Also when the successful answers are examined, the answers of the dyslexic group were considered by the jury to be more interesting and imaginative than those of the non-dyslexic ones (Fig. 5). This is very important because in a case of a drawing the jury compares the candidates according to whether or not they are interesting and imaginative.

As stated earlier, the different perceptions of dyslexic individuals appear to influence their potential achievements. In this study, it has been investigated whether or not these different perceptions could be considered advantages from the point of view of Communication Design. In light of the obtained data, we think that in order to improve the situation in the future, dyslexics could be encouraged to a special education program, especially in their childhood, of design education such as Communication.

According to the previous studies as mentioned above, even though it has been stated that dyslexics have tendencies especially in various fields of art, there have been no reported studies whether the potential talents of these individuals could be identified earlier. This study revealed that meaningful indications could be obtained from the tests conducted on dyslexics in their childhood.

We performed this study in order to find key points relevant to the field of communication. It is obvious that the number of the test participants could be increased in order to better understand the implications.

These experiments have been made by participants who registered in a single faculty of medicine. We propose that further study on dyslexics, who come from different regions and cultures and who speak different languages, could be more useful in the sense that it would provide more insight to the findings and possibly reveal more comprehensive results. Besides the position and the career histories of dyslexic individuals could also be followed after they are educated in the field of Communication Design.

## 7. Conclusion

'Dyslexic' individuals experience serious difficulties especially in their whole education process. The reasons for this brain-based problem are still a mystery. However, their existing situations could be considered differently in the case of communication design.

Table 3
Evaluation according to number of responds

| Parameter group | Test group | Control |
|---|---|---|
| Number of responses (out of 400 total questions) | 396 | 380 |
| Percentage of direction sign usage | 45 | 5 |
| Number of participants found successful for design education | 5 | 4 |
| The average time spent for the test (minutes) | 15 | 20 |

Everatt et al. [3] argued that the differences between the creativities of primary school children with and without dyslexia were small when it was compared to that of their adult counterparts. In other words his study shows that dyslexic adults tend to be more creative than non-dyslexic ones compared to primary school children. As the study of Everatt is considered, the differences between dyslexic and non-dyslexic primary school children in our study would have the possibility to become even more remarkable in adulthood.

We based our study on this point and examined 20 dyslexics with 20 control participants. The results are encouraging to think that dyslexic individuals could be an appropriate potential group of communication design education. However, after eliminating the limitations given in the discussion part, we may increase the effectiveness and validity of this pilot study.

## Acknowledgments

The authors would like to thank İnci Eviner, Ahmet Öktem, Asım Evren Yantaç Gary Berlind, Josh May, Owen Marmaduke, and Aziz Dursun. Particular thanks go to Beril Dilsizoğlu for unlimited help with and advice on this article.
